# An Evaluation of Birth Outcomes in Overweight and Obese Pregnant Women Who Exercised during Pregnancy

**DOI:** 10.3390/sports6040138

**Published:** 2018-11-03

**Authors:** Palee Myrex, Lorie Harper, Sara Gould

**Affiliations:** 1 School of Medicine, University of Alabama, Birmingham, AL 35233, USA; palee@uab.edu; 2 Department of Obstetrics and Gynecology, University of Alabama, Birmingham, AL 35233, USA; lmharper@uabmc.edu; 3 Department of Emergency Medicine/Department of Surgery–Orthopedics, University of Alabama, Birmingham, AL 35233, USA

**Keywords:** obesity, exercise, pregnancy, small for gestational age

## Abstract

It is well established that exercise has numerous health benefits, especially in regard to weight management for the obese and overweight population. However, there is limited data to support the safety or effects of exercise in the obese and overweight pregnant population despite the fact that exercise and weight management in this demographic is particularly important. In an effort to establish the safety profile of exercise during pregnancy in this population, we tested the hypothesis that exercise would not result in adverse birth outcomes. We surveyed postpartum women with an average BMI of 34.7 regarding their participation in exercise during pregnancy. Our primary outcome of interest was small for gestational age (SGA). Secondary outcomes included gestational age at delivery, mode of delivery, head circumference, length and birthweight as compared to those who did not exercise. SGA occurred in 12.5% of women who exercised in the first trimester compared to 14.9% in those who did not exercise (*p* = 0.678). Similar results were seen for women who exercised in the second and third trimesters. Intensity of exercise did not alter these findings and the analysis of secondary outcomes also did not demonstrate a difference between the groups. In conclusion, overweight and obese women who reported exercising during pregnancy did not have a higher incidence of SGA infants. Exercise should not be discouraged in pregnant women due to obesity.

## 1. Introduction

The benefits of exercise in the general population are widely acknowledged for having positive effects on overall health. This is especially true in the overweight and obese population as it has been well documented that exercise plays a key role in weight management and combating the associated metabolic disorders. However, there is limited data to support the effects of exercise on the overweight and obese pregnant population despite the fact that maternal obesity has important health implications for both mother and baby. The Centers for Disease Control (CDC) reports that maternal obesity is associated with chronic disease, cesarean section as mode of delivery, and elevated blood pressure during pregnancy [1]. Further, maternal obesity is the highest ranking modifiable risk factor for stillbirth in high-income countries contributing to approximately 8,000 stillbirths annually. Every 5-unit increase in maternal BMI increases the risk for stillbirth, neonatal death and infant death [2,3]. Infants born to obese mothers and mothers who gain more than the recommended amount of weight during pregnancy are at a higher risk for developing cardiovascular disease, hypertension, obesity and are more likely to have lower math, reading, and spelling scores [4,5,6].

Current exercise recommendations by the American Congress of Obstetricians and Gynecologists (ACOG) state that a woman with a low-risk pregnancy can participate in moderate exercise for ≥30 min per day on most, if not all, days of the week. However, ACOG also specifically comments that, “The information available in the literature is too limited to allow risk assignment for either premature labor or fetal growth restriction in recreational or professional athlete exercising mothers [7].” Currently, researchers have found inconsistent results regarding fetal weight and maternal exercise leading to the inability to make specific recommendations on the topic [8]. The aim of our study is to add to the pool of information and establish the safety profile of exercise during pregnancy in this specific population that stands to benefit a great deal from exercise and weight reduction. To our knowledge, this is the first study which takes into account various types, intensities, and timing of exercise to correlate with four different parameters of birth outcomes in the obese and overweight population.

## 2. Materials and Methods

Ethical approval for this study was obtained from The University of Alabama at Birmingham’s Institutional Review Board (ID: X160401002, approval granted on 17 May 2016). All women admitted to the postpartum floor of a public tertiary care university based hospital were screened for eligibility to participate. Patients were excluded from participating if they were under 16 years of age, had given birth to a stillborn, had given birth to a baby with severe birth defects and/or had given birth at less than 32 weeks gestation. Participants were also excluded if they were not overweight or obese as defined by the CDC. The CDC defines overweight as having a BMI of 25.0 to <30 and obese as having a BMI of 30 or greater. After screening and following informed consent, a total of 179 postpartum females were surveyed prior to hospital discharge regarding their participation in exercise during pregnancy. Each participant completed a 16-question survey specifying the type of exercise, frequency of exercise, duration of exercise, and perceived intensity of exercise during each trimester of pregnancy. Exercise type was divided into cardio (Ex: running, walking, swimming, biking, rowing, elliptical, etc.), resistance training (weight lifting, resistance bands, etc.) or yoga. Frequency of exercise was categorized as mild (1–2 days per week), moderate (2–4 days per week) or high (>4 days per week). Intensity was measured according to patient’s perceived physical exertion of mild, moderate, or intense.

Our primary outcome of interest was SGA as defined as being below the 10th percentile of birthweight for gestational age compared to a United States based birth weight cohort [9]. Secondary outcomes included gestational age at delivery, mode of delivery, birthweight, length & head circumference. Women who exercised in each trimester were compared to women who reported no exercise during pregnancy. Statistical analysis was completed using STATA^®^ (StataCorp LLC, College Station, TX, USA) with *p* <0.05 considered as statistically significant. All data were noted to be normally distributed (*p* >0.05). A two-sample t test with equal variances was conducted to compare birthweight as well as gestational age between those who exercised in the specified trimester and those who did not exercise during pregnancy. Fisher’s exact test was conducted to compare mode of delivery between those who exercised in the specified trimester and those who did not exercise during pregnancy. Finally, Pearson’s chi-squared test was used to compare SGA between those who exercised in the specified trimester and those who did not exercise. Pearson’s chi-squared test was also used to compare SGA in relation to type of exercise, intensity of exercise and frequency of exercise per trimester. The study was powered to detect a change in SGA from 10% to 25%, with an alpha of 0.05 and beta of 0.2. It was not powered to detect a smaller difference in SGA as the clinical significance of a smaller change is not clear.

## 3. Results

Participants’ average age was 26.5 years old with an average BMI of 34.7. The study population was predominately African American (69.3%) followed by White, (22.4%), Hispanic (7.8%) and Asian (0.6%). Of the 179 women surveyed, 27% (47) reported no exercise, 73% (128) reported exercise in the first trimester, 74% reported exercise in the second trimester (132), and 73% (129) reported exercise in the third trimester. Participant’s demographics are summarized below in Table 1.

### 3.1. Small for Gestational Age

Occurrence of SGA for each trimester is summarized below in Table 2. There was no significant difference between the exercise and no exercise groups.

Occurrence of SGA during each trimester in relation to different exercise types as compared to no exercise during pregnancy is summarized below in Table 3. There was no significant difference between the exercise and no exercise groups.

Occurrence of SGA during each trimester of exercise in relation to perceived intensity compared to no exercise during pregnancy is summarized below in Table 4. There was no significant difference between the exercise and no exercise groups.

Occurrence of SGA during each trimester of exercise in relation to frequency of exercise compared to no exercise during pregnancy is summarized below in Table 5. Frequency of exercise was categorized as mild (1–2 days per week), moderate (2–4 days per week) or high (>4 days per week). There was no significant difference between the exercise and no exercise groups.

### 3.2. Birthweight

Average birthweight for each trimester is summarized below in Table 6. There was no significant difference between the exercise and no exercise groups.

### 3.3. Head Circumference

Average head circumference for each trimester is summarized below in Table 7. There was no significant difference between the exercise and no exercise groups.

### 3.4. Length

Average length for each trimester is summarized below in Table 8. There was no significant difference between the exercise and no exercise groups when exercise occurred during the first and second trimester. When exercise occurred during the third trimester, results were significant for a shorter average body length.

### 3.5. Gestational Age

Average gestational age for each trimester is summarized below in Table 9. There was no significant difference between the exercise and no exercise groups.

### 3.6. Mode of Delivery

Mode of delivery assessed as cesarean section vs. non-cesarean for each trimester is summarized below in Table 10. From this data, a cesarean delivery occurred more often in women who exercised regardless of which trimester exercise occurred; however, these findings were not statistically significant as evidenced by the *p*-value.

## 4. Discussion

The purpose of this study was to examine the safety profile of exercise during pregnancy in the obese female. Our results confirm our hypothesis that exercise, regardless of trimester, type, or intensity, would not result in adverse birth outcomes.

Our primary outcome of interest was SGA which is defined as infants with a body weight below the 10th percentile for gestational age. When SGA is due to fetal growth restriction, it is associated with an increase in infant mortality and morbidity [10]. SGA and its complications have been a concern for the exercising mother as early studies suggested that women who exercise during pregnancy have smaller birthweight babies due to a decrease in infant total body fat stores [11]. On the contrary, a more recent meta-analysis demonstrated that structured prenatal exercise reduces the risk of having a large newborn without a change in the risk of having a small newborn [12]. It should be noted, however, that twenty-three of the twenty-eight trials analyzed in that study were low risk pregnancies and other studies examining specifically obese women have found that obese women who experienced gestational weight loss showed an increase in SGA and prematurity. These authors suggest that these risks outweigh the benefits of decreased pregnancy complications and advocate against gestational weight loss for obese women [13,14].

In our study of overweight and obese women who exercised during pregnancy, there was no increase in SGA compared to overweight and obese women who did not exercise during pregnancy. These findings were consistent regardless of which trimester exercise occurred in and regardless of what intensity the exercise was performed at. Likewise, the average birthweight was higher in the group that exercised, but not to the point of large for gestational age. These results were also consistent regardless of which trimester exercise was performed in. Other measures of intrauterine growth such as head circumference and length also did not appear to be adversely impacted by exercise. Body length of babies born to mothers who participated in exercise during their third trimester was shorter than the average body length of babies born to mothers who did not exercise; however, the shorter body length was still within the normal range. Our results are contrary to earlier literature which found that women who exercise have lower birthweight babies and to the studies showing increased premature birth rates in obese pregnant women who exercise [11,13]. Prematurity itself is a complication of maternal obesity and has been stated as a concern for obese women who participate in antenatal exercise but our results show that exercise did not increase this risk. Although our study did not show a decrease in prematurity in the exercise group relative to the non-exercise group, it does suggest that further studies examining exercise in the obese pregnant woman as a means to prevent premature births may be beneficial.

There was no statistically significant difference found in regard to type of exercise, frequency of exercise, or mode of delivery. In regard to mode of delivery, our data does approach significance to suggest a higher rate of cesarean delivery in the exercising population; however, it should be noted that all of the participants of this study were multiparous thus the higher cesarean rate may be due to repeat cesarean in patients with previous cesarean deliveries. Prior cesarean delivery was not a data point collected in our study and stands as a limitation of interpretation of this finding. Another limitation to this study that we acknowledge is the recall bias of participants about their exercise habits during pregnancy as surveys were administered to participants on the post-partum ward. Despite these limitations, our study strengthens the evidence against the concern for adverse fetal outcomes in obese women who exercise and argues for advocating exercise in the obese pregnant female.

## 5. Conclusions

Obese women who reported exercising during pregnancy did not have a higher incidence of SGA infants compared to obese women who did not exercise during pregnancy. Exercise should not be discouraged in obese women due to pregnancy.

## Figures and Tables

**Table 1:** Demographics.

Demographics	Exercise (SD) or (n)	No Exercise (SD) or (n)	*p*-Value
**Age**	26.0 years (5.9 years)	27.75 years (5.8 years)	0.0872
**BMI**	35.7 (9.3)	33.7 (7.1)	0.1814
**Nulliparous**	0%	0%	
**Race**			
*Black*	70.7% (93/132)	66.0% (31/47)	0.191
*White*	20.5% (27/132)	27.7% (13/47)	0.191
*Hispanic*	9.1% (12/132)	4.3% (2/47)	0.191
*Asian*	0% (0/132)	2.1% (1/47)	0.191
**Infant Sex**			
*Male*	47.7% (63/132)	57.5% (27/47)	0.252
*Female*	52.3% (69/132)	42.5% (20/47)	0.252

**Table 2:** SGA per trimester.

Trimester	Exercise (n)	No Exercise (n)	*p*-Value
1st Trimester	12.50% (16/128)	14.89% (7/47)	0.678
2nd Trimester	12.12% (16/132)	14.89% (7/47)	0.626
3rd Trimester	13.95% (18/129)	14.89% (7/47)	0.874

**Table 3:** SGA per exercise type during each trimester.

Trimester	No Exercise	Cardio	*p*-Value	Resistance	*p*-Value	Yoga	*p*-Value
1st Trimester	14.89% (7/47)	13.11% (16/122)	0.763	9.09% (2/22)	0.505	12.50% (2/16)	0.813
2nd Trimester	14.89% (7/47)	12.31% (16/130)	0.651	9.52% (2/21)	0.546	15.38% (2/13)	0.965
3rd Trimester	14.89% (7/47)	14.52% (18/124)	0.950	13.33% (2/15)	0.881	18.18% (2/11)	0.786

**Table 4:** SGA per intensity of exercise during each trimester.

Trimester	No Exercise	Mild	Moderate	High	*p*-Value
1st Trimester	14.89% (7/47)	0.00% (0/9)	11.63% (10/86)	20.00% (5/25)	0.443
2nd Trimester	14.89% (7/47)	0.00% (0/12)	11.70% (11/94)	20.83% (5/24)	0.336
3rd Trimester	14.89% (7/47)	5.88% (1/17)	14.47% (11/76)	18.18% (6/33)	0.708

**Table 5:** SGA per frequency of exercise during each trimester.

Trimester	No Exercise	1–2 Days	2–4 Days	>4 Days	*p*-Value
1st Trimester	14.89% (7/47)	9.09% (1/11)	7.46% (5/67)	20.00% (10/50)	0.237
2nd Trimester	14.89% (7/47)	0.00% (0/11)	11.43% (8/70)	15.69% (8/51)	0.514
3rd Trimester	14.89% (7/47)	9.09% (2/22)	15.28% (11/72)	14.29% (5/35)	0.906

**Table 6:** Birthweight.

Trimester	Exercise (SD)	No exercise (SD)	*p*-Value
1st Trimester	3006.10 g (641.68 g)	3067.21 g (634.71 g)	0.5762
2nd Trimester	2973.95 g (644.39 g)	3067.21 g (634.71 g)	0.3935
3rd Trimester	2996.75 g (624.21 g)	3067.21 g (634.71 g)	0.5104

**Table 7:** Head Circumference.

Trimester	Exercise (SD)	No Exercise (SD)	*p*-Value
1st Trimester	33.33 cm (2.22 cm)	33.51 cm (2.08 cm)	0.6277
2nd Trimester	33.22 cm (2.22 cm)	33.51 cm (2.08 cm)	0.4306
3rd Trimester	33.31 cm (2.22 cm)	33.51 cm (2.08 cm)	0.5937

**Table 8:** Length.

Trimester	Exercise (SD)	No Exercise (SD)	*p*-Value
1st Trimester	19.64 in (3.08 in)	20.69 in (6.23 in)	0.1408
2nd Trimester	19.57 in (3.09 in)	20.69 in (6.23 in)	0.1132
3rd Trimester	19.44 in (1.40 in)	20.69 in (6.23 in)	0.0332

**Table 9:** Gestational Age.

Trimester	Exercise (SD)	No Exercise (SD)	*p*-Value
1st Trimester	38.03 weeks (2.43 weeks)	37.95 weeks (2.02 weeks)	0.8431
2nd Trimester	37.98 weeks (2.51 weeks)	37.95 weeks (2.02 weeks)	0.9442
3rd Trimester	38.14 weeks (2.49 weeks)	37.95 weeks (2.02 weeks)	0.6349

**Table 10:** Cesarean Delivery.

Trimester	Exercise	No Exercise	*p*-Value
1st Trimester	41.41% (53/128)	29.79% (14/47)	0.161
2nd Trimester	41.67% (55/132)	29.79% (14/47)	0.151
3rd Trimester	36.43% (47/129)	29.79% (14/47)	0.412

## References

[1] Center for Disease Control and Prevention. https://www.cdc.gov/prams/pdf/snapshot-report/nutritionpaandobesity.pdf.

[2] Flenady V., Koopmans L., Middleton P., Frøen J.F., Smith G.C., Gibbons K., Coory M., Gordon A., Ellwood D., McIntyre H.D. (2011). Major risk factors for stillbirth in high-income countries: A systematic review and meta-analysis. Lancet.

[3] Aune D., Saugstad O.D., Henriksen T., Tonstad S. (2014). Maternal body mass index and the risk of fetal death, stillbirth, and infant death: A systematic review and meta-analysis. JAMA.

[4] Perng W., Gillman M.W., Mantzoros C.S., Oken E. (2014). A prospective study of maternal prenatal weight and offspring cardiometabolic health in mid-childhood. Ann. Epidemiol..

[5] Gaillard R. (2015). Maternal obesity during pregnancy and cardiovascular development and disease in the offspring. Eur. J. Epidemiol..

[6] Pugh S.J., Hutcheon J.A., Richardson G.A., Brooks M.M., Himes K.P., Day N.L., Bodnar L.M. (2016). Child academic achievement in association with pre-pregnancy obesity and gestational weight gain. J. Epidemiol. Community Health.

[7] Artal R., O’Toole M. (2003). Guidelines of the American College of Obstetricians and Gynecologists for exercise during pregnancy and the postpartum period. Br. J. Sports Med..

[8] Kramer M.S., McDonald S.W. (2006). Aerobic exercise for women during pregnancy. Cochrane Database Syst. Rev..

[9] Duryea E.L., Hawkins J.S., McIntire D.D., Casey B.M., Leveno K.J. (2014). A revised birth weight reference for the United States. Obstet. Gynecol..

[10] Liu J., Wang X.F., Wang Y., Wang H.W., Liu Y. (2014). The incidence rate, high-risk factors, and short- and long-term adverse outcomes of fetal growth restriction: A report from Mainland China. Medicine (Baltim.).

[11] Clapp J.F., Capeless E.L. (1990). Neonatal morphometrics after endurance exercise during pregnancy. Am. J. Obstet. Gynecol..

[12] Wiebe H.W., Boule N.G., Chari R., Davenport M.H. (2015). The effect of supervised prenatal exercise on fetal growth: A meta-analysis. Obstet. Gynecol..

[13] Beyerlein A., Schiessl B., Lack N., von Kries R. (2011). Associations of gestational weight loss with birth-related outcome: A retrospective cohort study. BJOG.

[14] Kapadia M.Z., Park C.K., Beyene J., Giglia L., Maxwell C., McDonald S.D. (2015). Weight loss instead of weight gain within the guidelines in obese women during pregnancy: A systematic review and meta-analyses of maternal and infant outcomes. PLoS ONE.

